# Waterhole detection using a vegetation index in desert bighorn sheep (*Ovis canadensis cremnobates*) habitat

**DOI:** 10.1371/journal.pone.0211202

**Published:** 2019-01-22

**Authors:** Jonathan Gabriel Escobar-Flores, Sarahi Sandoval, Raul Valdez, Eahsan Shahriary, Jorge Torres, Sergio Alvarez-Cardenas, Patricia Gallina-Tessaro

**Affiliations:** 1 Instituto Politécnico Nacional, Centro Interdisciplinario De Investigación para el Desarrollo Integral Regional, Unidad Durango, Durango, México; 2 CONACYT-Instituto Politécnico Nacional, Centro Interdisciplinario De Investigación para el Desarrollo Integral Regional, Unidad Durango, Durango, México; 3 Department of Fish, Wildlife and Conservation Ecology, New Mexico State University, Las Cruces, New Mexico, United States of America; 4 Environmental Science and Engineering Program, University of Texas at El Paso, El Paso, Texas, United States of America; 5 Departament of Computer Science, Centro de Investigación Científica y de Educación Superior de Ensenada, Ensenada, Baja California, México; 6 Centro de Investigaciones Biológicas del Noroeste, La Paz, Baja California Sur, México; Universitat Autonoma de Barcelona, SPAIN

## Abstract

In arid ecosystems, desert bighorn sheep are dependent on natural waterholes, particularly in summer when forage is scarce and environmental temperatures are high. To detect waterholes in Sierra Santa Isabel, which is the largest area of desert bighorn sheep habitat in the state of Baja California, Mexico, we used the normalized difference vegetation index (NDVI) and normalized difference water index (NDWI) from Sentinel-2 satellite images. Waterhole detection was based on the premise that sites with greater water availability, where NDVI was higher, can be identified by their density of vegetation greenness. For the detected waterholes, we estimated the escape terrain (presence of cliffs or steep, rocky slopes) around each by the vector ruggedness measure to determine their potential use by desert bighorn sheep based on the animals’ presence as documented by camera traps. We detected 14 waterholes with the NDVI of which 11 were known by land owners and 3 were unrecorded. Desert bighorn were not detected in waterholes with high values of escape terrain, i.e., flat areas. Waterhole detection by NDVI is a simple method, and with the assistance and knowledge of the inhabitants of the Sierra, it was possible to confirm the presence each waterhole in the field.

## Introduction

According to Seager et al. [1] the arid regions of southwestern North America will be affected by hydroclimate, they estimated a reduction in mean annual precipitation up to 0.1 mm/day, which will exacerbate dry conditions in the twenty-first century. Also, Lioubimtseva [2] estimated mean annual temperature increases in the north of Mexico to be about 2 °C. The main effects will be a reduction in the availability of forage and waterholes [3].

Desert bighorn sheep (*Ovis canadensis cremnobates*) are well-adapted to arid environments because of metabolic adaptations that enable the bighorn to inhabit deserts such as those in the southwestern United States [4] and in San Felipe in Baja California [5], but they also drink surface water [6]. Knowing the locations of surface water can aid in conservation, but the presence of a waterhole does not ensure it will be used. Other components of habitat can influence the use of waterholes by desert bighorn sheep. For example, if water is within 20 meters of escape terrain [7] and vegetation cover is less than 25–30% [8], there is a higher probability that desert bighorn sheep will use the waterhole [9,10]. Escape terrain refers to the presence of cliffs or steep, rugged terrain and rocky slopes where desert bighorn can outmanoeuvre predators [11].

There has been much effort to study surface water detection by remote sensing through spectral near/mid-infrared bands, which have strong water-detecting features [12]. From these bands, algorithms have been developed to detect water, referring to mathematical models that enhance water signals for a given pixel in images obtained from visible/near-infrared scanning sensors [13]. The most used water algorithms are the normalised difference vegetation index (NDVI), the normalised difference water index (NDWI) [14, 15] and the modified normalised difference water index (MNDWI), which is only used to detect waterholes larger than 400 m^2^ [16, 17].

We used the NDWI and NDVI for waterhole detection because these algorithms use spectral bands with 10-meter spatial resolution, which detects waterholes with areas of 100 m^2^, increasing the detection of waterholes in comparison to the MNDWI. The NDVI is useful for detecting waterholes because in arid ecosystems higher NDVI will represent areas of greener vegetation, and should therefore represent areas of greater surface water availability [18,19, 20]. Also, the NDVI has positive correlation with plant cover and biomass productivity [21, 22, 23].

The objectives of this study were to compare the abilities of NDVI and NDWI for waterhole detection in the wet and dry seasons by working with local guides to ground-truth waterholes detected with NDVI and NDWI, and to evaluate the use of accurately detected waterholes by desert bighorn sheep in both the wet and dry seasons using camera traps.

## Materials and methods

The individuals in this manuscript has been given written informed consent (as outlined in PLOS consent form) to publish these case details.

No permits were required, because data were analysed with satellite images.

### Study area

**Fig 1:**
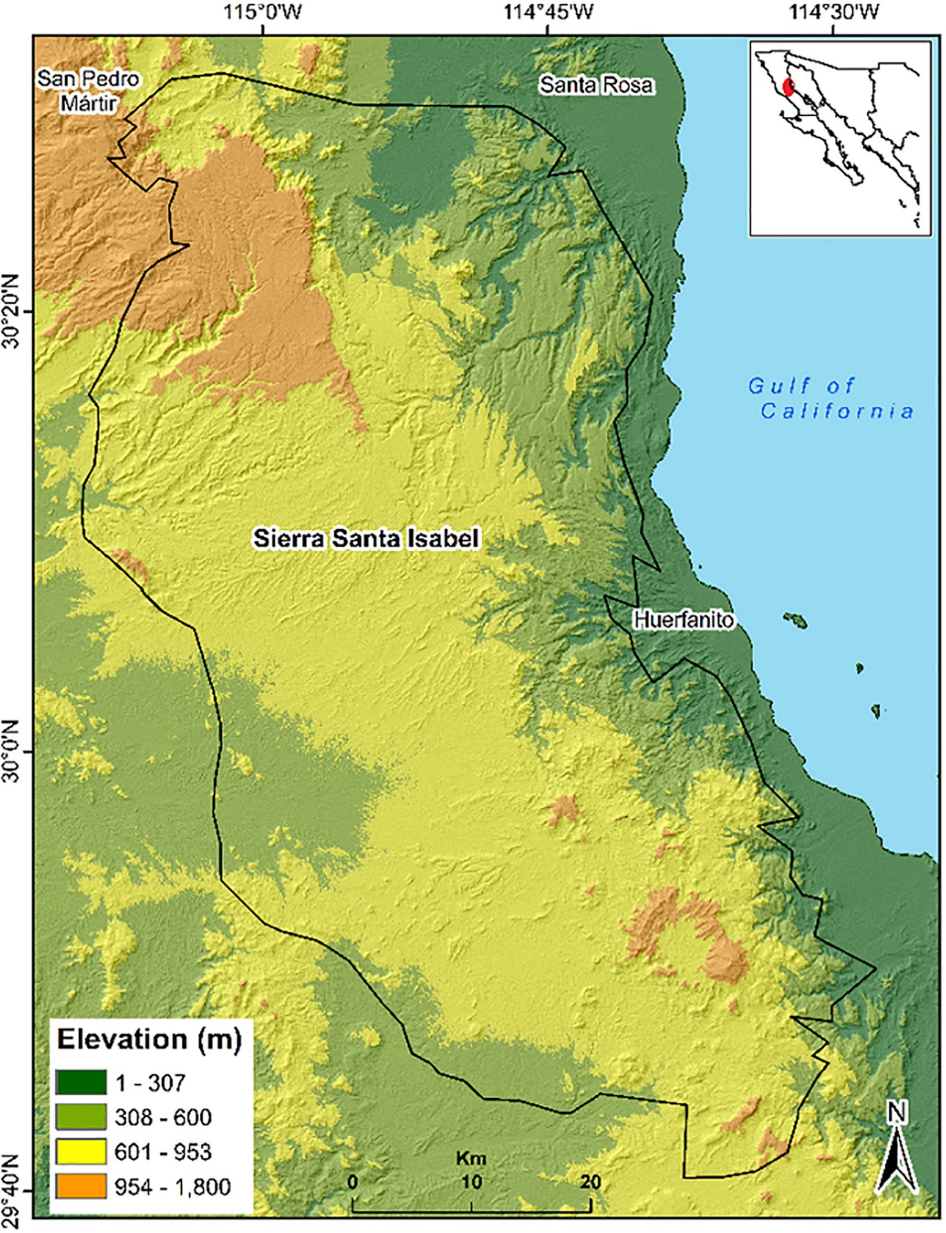
Location of Sierra Santa Isabel, Baja California, Mexico.

The Sierra Santa Isabel mountain range is located in the central region of the state of Baja California, Mexico (Fig 1). The Sierra is characterized as having the largest continuous habitat for desert bighorn sheep (*Ovis canadensis cremnobates*) in the state (2,072.45 km^2^), with an estimated population of 300–400 wild sheep [24, 25, 26]. The Sierra is situated in two phytogeographic regions of the Sonoran Desert; namely, the Colorado Desert and the Central Desert. The Colorado Desert includes the valleys and hills close to the Gulf of California at elevations of 100 m to 400 m with predominant vegetation of *Larrea tridentata*, *Fouquieria splendens*, *Psorothamnus spinosus* and *Olneya tesota*. The mean precipitation during the wet season in 2015 (beginning in November and ending in February) was 30 mm ± 5.8 with a maximum temperature of 25 °C. The precipitation in the dry season (from May to July) was 0.4 mm ± 0.02 with a maximum temperature of 40 °C.

The Central Desert occupies the largest part of the Sierra at elevations from 400 m to 1680 m and its topography is mainly canyons and elevations that exceed 1600 m. The annual mean precipitation was 100 mm in 2015. The wet and dry seasons are the same as for the Colorado Desert [27]. The main plant species are *Ambrosia dumosa*, *Bursera microphylla*, *Fouquieria columnaris*, *Cercidium microphyllum* and *Pachycereus discolor* [28].

### Detecting waterholes

To evaluate NDVI and NDWI as viable tools for detecting waterholes in desert bighorn sheep habitat, we obtained and processed four satellite images from Sentinel-2A with the multispectral sensor instrument (MSI), which covered the entire Sierra. The spatial resolution (10 m per pixel) is three times greater than that of Landsat images, thus increasing the potential for waterhole detection [16]. The dataset acquired on 3 February 2015 represents the end of the wet season, and the 2 July 2015 dataset represents the end of the dry season [27]. These two seasons were chosen to compare the effect of seasonality on waterhole detection. Four images for each dataset were downloaded from the US Geological Survey (USGS) Global Visualization Viewer server [http://glovis.usgs.gov], which corresponded to the trajectory codes T11RPP for the Colorado Desert and T11RQP for the Central Desert in the study area (S1 Appendix). We used four satellite images. Other studies that detected water with finer resolution data (< 10 m) used two to four images [15, 16].

From the satellite imagery, NDVI and NDWI were used for waterhole detection. NDVI utilises the absorption proportions in the red (R) region and vegetation cover reflectance in the near-infrared (NIR) region [18]. NDVI was calculated with the following equation: NDVI = NIR−R/NIR + R. The NDVI values ranged from −1.0 to 1.0, In the Sierra the values of NDVI < 0.05 indicated scarce vegetation [20]. We used a threshold of NDVI > 0.19 for detecting waterholes. The NDVI was estimated for both the wet and dry seasons. Three bands (RGB: band8/NDVI/band2) were combined to highlight sites that had apparent waterholes, and their UTM coordinates were obtained.

NDWI maximizes waterhole reflectance in the green band and minimizes water reflectance in the NIR band. NDWI was calculated with bands three and eight of the Sentinel 2A- MSI sensor. Waterhole identification by NDWI has been controversial; some studies have indicated that soil brightness affects the index results or confuses waterholes with shaded areas [15]. NDWI values > 0.10 indicated a probable waterhole [14]. To visualise waterhole detection by NDWI, a combination band analysis was performed among the following bands, RGB: band4/band3/NDWI.

### Evaluation of the success of NDVI and NDWI

We created a map of waterholes detected by NDVI and NDWI, and showed it to local land owners who manage federally approved Sustainable Wildlife Management Units. With the assistance of these local land owners, we collected field data one week after processing the satellite images, and waterhole locations were recorded with a GPS and verified in the field in February—March 2015 for 10 days. At each site, we recorded the plant species that were visually abundant around the edge of the waterhole following the estimation—appreciation method using 500 meters as the buffer (perimeter) value [29].

### Presence of wildlife at the waterholes

Infrared-triggered cameras (LLC Grand and Prairie, 9504, Stealth cam, Texas, USA; Overland Park, 9200, Bushnell Trophy Cam, Kansas City, USA) were placed at each identified waterhole [30, 31]. These infrared-triggered cameras were used to document use of the waterhole by desert bighorn sheep and other species of wildlife. We programmed a setting with a 5-min delay between consecutive events (photographs) and captured three photos per event. The photo sampling periods covered February to September 2015.

### Escape terrain

By means of a digital terrain model (DTM) with a 15-m pixel resolution [http://www.inegi.org.mx], the vector ruggedness measure (VRM) was calculated for each of the identified waterholes. VRM values range from 0 for flat areas to 1 for canyons and ravines. The values of this index provide a reliable approximation of escape terrain for bighorn habitat [32]. The VRM incorporates slope and aspect heterogeneity using tri-dimensional dispersion of normal vectors. The DTM and VRM were processed with Qgis software [33].

### Statistical analysis

Waterhole data were analyzed with one-way ANOVA, and we assumed a random sample. The regression was calculated using SAS/STAT software [34]. We used means of VRM values within a 500-m radius because Alvarez-Cardenas [9] observed a greater number of desert bighorn sheep in Sierra Del Mechudo at this radius. This Sierra has topographic characteristics similar to Sierra Santa Isabel. Each detected waterhole was compared using the Fisher LSD test [35]. The studentized residual was calculated and checked using the Kolmogorov-Smirnov test [36]. The homogeneity of variance was tested using Levene’s test [37]. To determine if the averages of NDVI and NDWI varied between wet and dry seasons first we calculated values within a 500-m radius of each detected waterhole. NDVI variations were then compared using the paired t-test. We conducted the same analysis for the NDWI, using the SAS software [35, 37]. The alpha level was set at 0.05 and the p-value cutoff was < 0.05. The differences between waterholes according to NDVI and NDWI were graphed with box diagrams.

The efficacy of NDVI and NDWI waterhole detection was evaluated using the Kappa (*k*) coefficient [38, 39], with a 2 × 2 table with the following considerations: i) positive agreement between the number of waterholes detected by the algorithms (NDVI or NDWI) and local land owners, ii) waterholes detected that do not exist according to the landowners iii) waterholes not detected and that do exist iv) both correct absences, where waterholes were not present and were not detected.

The standard error (SE) of *k* for a 2 × 2 table was estimated with the following equation:

$$SE=\sqrt{\frac{Po\;(1-Po)}{n\;(1-Pe)²}}$$

where *Po* = represents the observed agreement, *Pe* = represents chance agreement, and n is the total number of observations. The 95% confidence intervals (CIs) of *k* were calculated as follows: CI _95%_ = *k* ± 1.96 · SE (*k*). The parameter *k* takes on values between 0 and 1; *k* close to 1 indicate a greater degree of agreement between classification and observation, and a value of 0 suggests that the agreement is random.

## Results

### Waterhole detections

**Fig 2:**
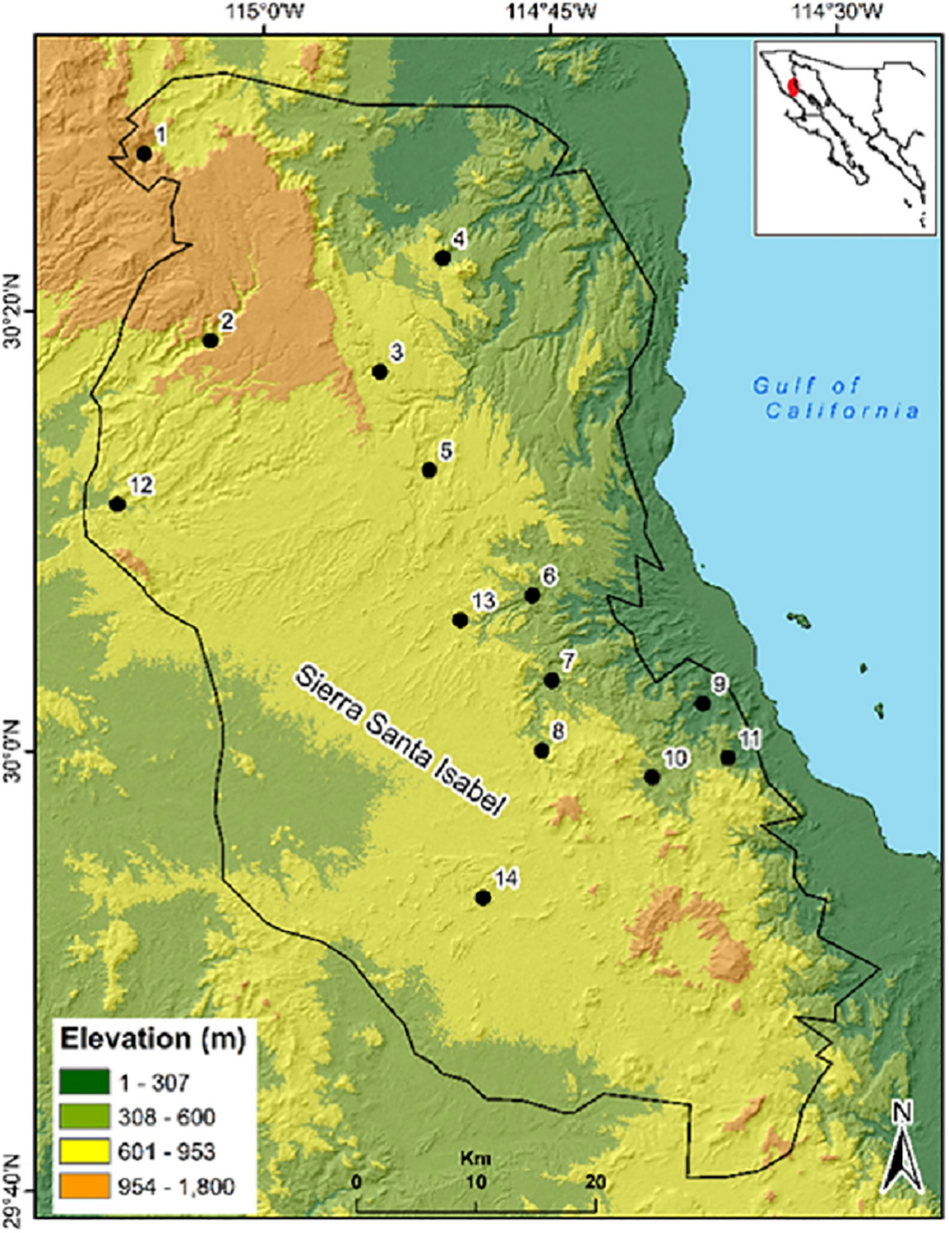
Location of waterholes in Sierra Santa Isabel, Baja California. 1. Matomi, 2. Grande, 3. Las Blancas, 4. Canelo, 5. Hemes, 6. Zamora, 7. Cordero, 8. Volcán, 9. Miramar, 11. Palmito, 12. San Agustín, 13. Peralta, 14. Dulce.

Within the Sierra we detected 14 waterholes in the wet season and 12 waterholes in the dry season with NDVI > 0.19. All waterholes were confirmed in the field (Fig 2). Waterhole detection by this method is viable independent of the season of the year. NDVI values higher than 0.4 have been associated with sites with vegetation cover greater than 40%. Only the Canelo waterhole had NDVI values < 0.20. Other sites had NDVI values > 0.20 but these waterholes are located in flat sites more than 30 km from the nearest known desert bighorn sheep population, therefore these were discarded. When the NDWI was calculated in the dry season, only four waterholes were detected. Waterhole detection with NDWI values greater than 0.06 increased during the wet season (Table 1).

**Table 1:** Seasonal NDVI and NDWI; the maximum value of the pixel where the waterhole was detected. Bold type indicates waterholes detected with NDWI. Seasons were wet (November to February) and dry (May to July). The sizes of the waterholes were estimated in the wet season, area is in m^2^.

Waterhole	NDVI		NDWI		Surface area (m^2^)
	Dry	Wet	Dry	Wet	
Matomi	0.54	0.51	−0.13	−0.27	393.95
Grande	0.42	0.42	**0.14**	−0.18	1,068.2
Zamora	0.59	0.51	−0.02	**0.15**	2,000.2
Hemes	0.17	0.23	−0.09	**0.06**	90.5
Blancas	0.20	0.28	−0.13	−0.26	661.3
Canelo	0.13	0.31	−0.06	**0.14**	139.2
Cordero	0.22	0.29	**0.029**	**0.40**	193.9
Azul	0.52	0.49	−0.008	**0.20**	808.2
Volcán	0.47	0.39	−0.005	**0.19**	195.4
Miramar	0.28	0.31	**0.092**	**0.36**	325.7
Palmito	0.43	0.57	**0.35**	**0.35**	1,194.1
San Agustín	0.35	0.21	−0.14	−0.27	485.2
Peralta	0.34	0.29	−0.06	−0.07	739.1
Dulce	0.40	0.41	−0.12	−0.12	485.2

#### Waterhole evaluation

**Fig 3:**
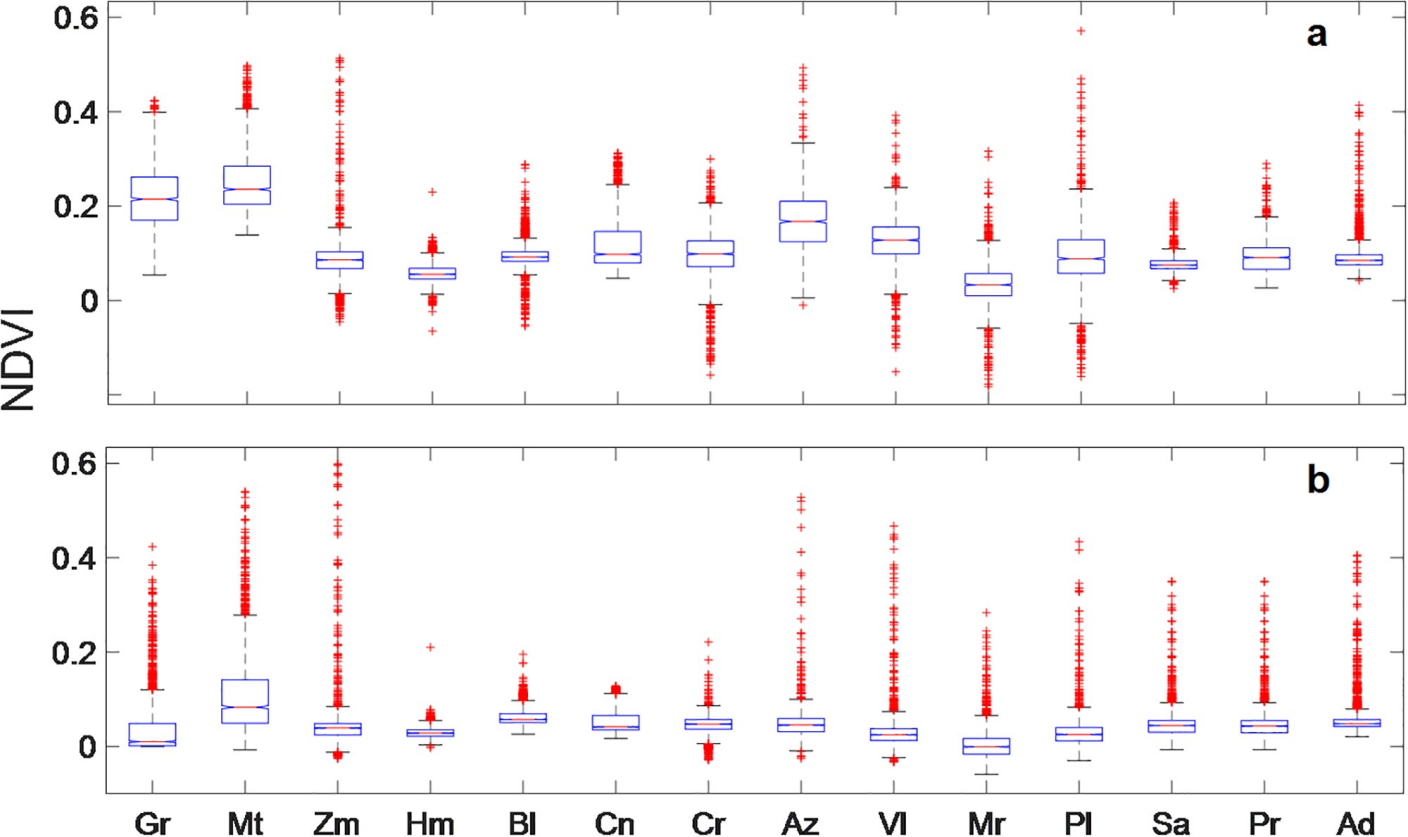
Comparison of NDVI values at waterholes detected during a) wet season (November to February), and b) dry season (May to July) in Sierra Santa Isabel, Baja California, Mexico. Gr = Grande, Mt = Matomi, Zm = Zamora, Hm = Hemes, Bl = Blancas, Cn = Canelo, Cr = Cordero, Az = Zamora, Vl = Volcán, Mr = Miramar = Pl = Palmito, Sa = San Agustín, Pr = Peralta, Ad = Dulce.

Waterhole NDVI values varied from 0.23 to 0.57 for the wet season and 0.13 to 0.54 for the dry season. Based on the ANOVA analysis, the average NDVI values within the 500-m radius differed seasonally (dry season, Fisher’s LSD-test = 590.42, p = 0.0001; and wet season, Fisher’s LSD-test = 297.62, p = 0.0001). The paired t-test confirmed that the average NDVI (i.e., NDVI > 0.09) values were greater in the wet season than the dry season at all waterholes (Table 2; Fig 3a and 3b). The value of *k* obtained for the NDVI was the same for both seasons; *k* (95% CI) = 0.67 (0.40–0.93).

**Table 2:** Comparison of seasonal (wet = November to February; dry = May to July) NDVI, minimum and maximum value of pixel around the edge of the waterhole using paired t-test at waterholes detected in Sierra Santa Isabel, Baja California, Mexico.

Waterholes	NDVI wet			NDVI dry			T-Student	P Values
	Min	Max	Average	Min	Max	Average		
Matomi	0.13	0.51	0.25	−0.07	0.54	0.10	97.51	0.0001***
Grande	0.05	0.42	0.21	0.01	0.42	0.02	70.09	0.0001***
Zamora	−0.04	0.51	0.08	−0.02	0.59	0.04	23.89	0.0001***
Hemes	−0.06	0.23	0.05	−0.02	0.17	0.02	54.91	0.0001***
Blancas	−0.05	0.28	0.09	0.02	0.20	0.06	44.36	0.0001***
Canelo	0.04	0.31	0.11	0.01	0.13	0.05	55.30	0.0001***
Cordero	−0.15	0.29	0.09	−0.02	0.22	0.04	39.87	0.0001***
Azul	−0.01	0.49	0.17	−0.02	0.52	0.05	75.03	0.0001***
Volcán	−0.15	0.39	0.12	−0.03	0.47	0.03	60.64	0.0001***
Miramar	−0.18	0.31	0.03	−0.05	0.28	0.04	25.08	0.0001***
Palmito	−0.16	0.57	0.09	−0.03	0.43	0.02	38.65	0.0001***
San Agustín	0.02	0.21	0.07	−0.06	0.35	0.04	37.40	0.0001***
Peralta	0.02	0.29	0.09	−0.06	0.34	0.04	86.10	0.0001***
Dulce	0.04	0.41	0.09	0.02	0.40	0.05	71.25	0.0001***

*** (P<0.001)

**Fig 4:**
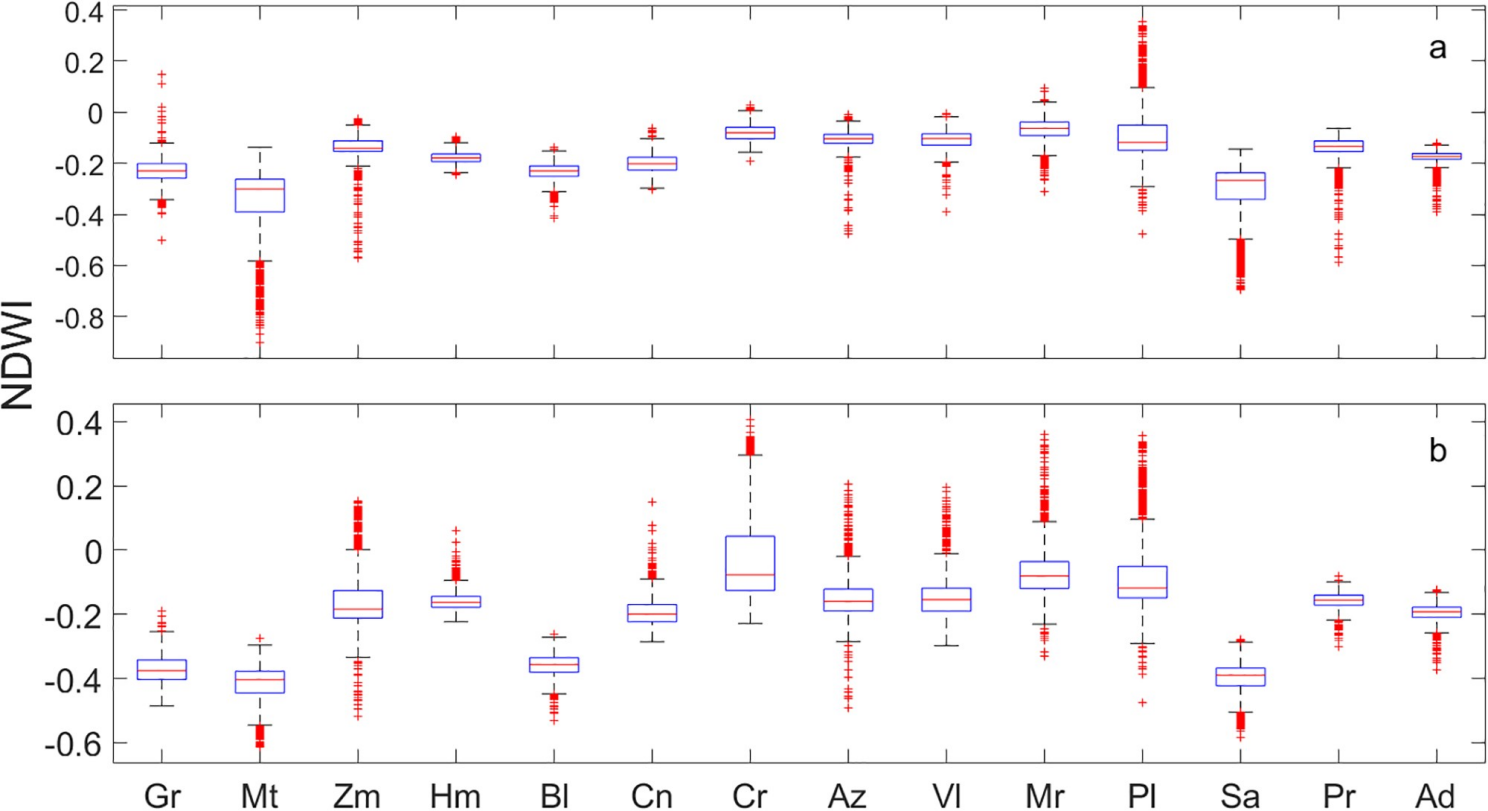
Comparison of NDWI values at waterholes detected during a) the wet season (November to February), and b) dry season (May to July) in Sierra Santa Isabel, Baja California, Mexico. Gr = Grande, Mt = Matomi, Zm = Zamora, Hm = Hemes, Bl = Blancas, Cn = Canelo, Cr = Cordero, Az = Zamora, Vl = Volcán, Mr = Miramar = Pl = Palmito, Sa = San Agustín, Pr = Peralta, Ad = Dulce.

The NDWI values were not significantly different for any waterhole, with the exception of the Miramar waterhole (Table 3); this waterhole was detected during both seasons. All the waterholes had negative average NDWI values within a 500-m radius. We used the maximum values of NDWI to detect waterholes during both seasons, but NDWI values were not as accurate as NDVI values (Table 3). Of the larger waterholes, only Palmito and Miramar were detected by NDWI in both seasons (1,194.1 m^2^) (Fig 4a and 4b). The values for *k* obtained for the NDWI in the dry season were *k* (95% CI) = 0.126 (−0.24–0.49) and in the wet season *k* (95% CI) = 0.27 (−0.36–0.59).

**Table 3:** Comparison of NDWI values during the wet (November to February) and dry (May to July) seasons using paired t-test in Sierra Santa Isabel, Baja California, Mexico.

Waterholes	NDWI wet			NDWI dry			T-Student	P value
	Min	Max	Average	Min	Max	Average		
Matomi	−0.48	-0.27	−0.37	−0.50	−0.13	−0.23	97.95	0.0001***
Grande	−0.61	-0.18	−0.42	−0.90	0.14	−0.35	29.40	0.0001***
Zamora	−0.51	0.15	−0.16	−0.57	−0.02	−0.13	17.93	0.0001***
Hemes	−0.22	0.06	−0.16	−0.24	−0.09	−0.18	-30.74	0.0001***
Blancas	−0.53	-0.26	−0.36	−0.42	−0.13	−0.23	150.98	0.0001***
Canelo	−0.28	0.14	−0.19	−0.30	−0.06	−0.20	-10.56	0.0001***
Cordero	−0.22	0.40	−0.02	−0.19	0.02	−0.08	-19.32	0.0001***
Azul	−0.49	0.20	−0.15	−0.48	−0.00	−0.11	34.81	0.0001***
Volcán	−0.29	0.19	−0.15	−0.39	−0.005	−0.11	33.80	0.0001***
Miramar	−0.32	0.36	−0.07	−0.31	0.092	−0.07	1.60	0.108 ns
Palmito	−0.46	0.35	−0.09	−0.47	0.35	−0.09	3.63	0.0003***
San Agustín	−0.58	−0.27	−0.40	−0.69	−0.14	−0.30	37.85	0.0001***
Peralta	−0.30	−0.07	−0.16	−0.59	−0.06	−0.14	24.68	0.0001***
Dulce	−0.37	−0.12	−0.20	−0.39	−0.12	−0.18	36.33	0.0001***

(P<0.001): ***. ns: non-significant

#### Escape terrain

**Fig 5:**
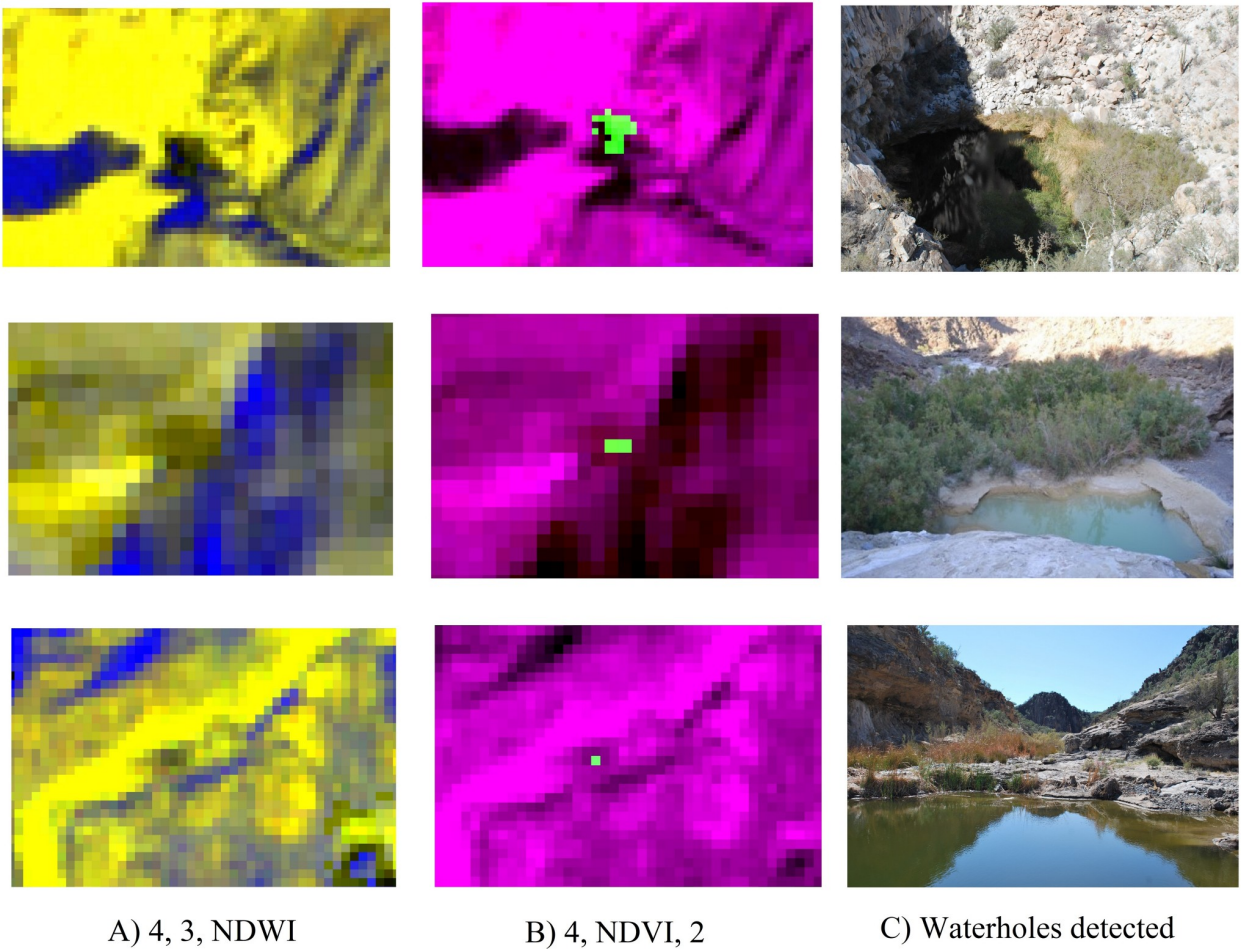
The band combinations used for detecting waterholes in Sierra Santa Isabel, Baja California, Mexico. A) waterholes detected by NDVI but not detected by NDWI (pixels in blue); B) waterholes detected only by the NDVI are shown in pixels in green; C) confirmed waterholes from top to bottom: Zamora, Azul and Volcán.

Desert bighorn sheep were detected at only 11 waterholes. The waterholes at which the greatest numbers of desert bighorn were photographed were at Arroyo Grande (140) and El Zamora (120) (Fig 5, Table 4). The escape terrain differed between waterholes (Fishers LSD-test = 117.92, p = 0.0001 (Table 4). No photographs of desert bighorn sheep were obtained at the Agua Dulce, Peralta, or San Agustin waterholes.

**Table 4:** Escape terrain values (mean ± SE; P < 0.05) at waterholes in Sierra Santa Isabel, Baja California, Mexico. Means with different letters are statistically different based on Fisher’s LSD multiple comparison test.

Waterholes	Single	Escape terrain (Mean ± SE)
Zamora	120	0.0100±0.0004 a
Cordero	67	0.0098±0.0003 a
Miramar	18	0.0094±0.0005 ab
Palmitas	12	0.0089±0.0004 bc
Azul	42	0.0085±0.0003 cd
Blancas	8	0.0080±0.0004 cd
Grande	140	0.0079±0.0003 d
Volcán	44	0.0062±0.0003 e
Matomi	72	0.0051±0.0002 f
Canelo	39	0.0042±0.0003 g
Hemes	29	0.0029±0.0001 h
San Agustín	0	0.0010±0.0004 i
Peralta	0	0.0007±0.0004 i
Agua dulce	0	0.0005±0.0002 i

A total effort of 3,380 camera trap sampling days was made at the fourteen waterholes, which yielded 1820 images of 12 wild mammals, 21 birds, 1 amphibian, and 2 reptiles. The month with the most photographic records of wildlife was July, which accounted for 50% of the total records (S3 Appendix). Three photographs were new records of *Vulpes macrotis*, *Bassariscus astutus* and *Patagioneas fasciata*. The waterholes with most pictures of desert bighorn sheep were Grande with 140 images and Zamora with 120. At four waterholes (Zamora, Cordero, Volcán and Azul) the mountain lion (*Puma concolor*) was photographed.

## Discussion

Waterhole detection was found to be accurate using NDVI. This finding has previously been reported for arid environments such as the Chad Desert [40], and at Lake Urmia [41]. The threshold of NDVI > 0.19 was useful, as it detected fourteen waterholes in the wet season, but in the dry season the threshold changed; the Hemes and Cordero waterholes had values of 0.17 and 0.13 respectively. These waterholes were detected because there is a contrast with neighboring NDVI values that were less than 0.05 [7]. In summary, twelve waterholes were springs and two were ephemeral.

NDWI was not as accurate as the NDVI for waterhole detection. For some waterholes that were previously identified by NDVI, the values of NDWI were found to be negative. According to McFeeters [14], NDWI values less than 0 are associated with bright surfaces without the presence of water. We propose two possible reasons why NDWI might not detect water; i) shiny surfaces of limestone rocks, quartz conglomerates; these rocks were present at some waterholes, for example the Matomi, Peralta, and Dulce waterholes; ii) the size of the waterhole surface in the dry season, for example the Hemes and Canelo waterholes (these are the smallest; Table 1) and in the dry season hardly have any surface water.

The threshold to find waterholes with NDWI was 0.029 in the dry season and 0.06 in the wet season. These thresholds contrast with reports of research conducted near cities [14, 15] where it is noted that thresholds below 0.3 can confuse waterholes with urban infrastructure; however, this did not happen in the present study because there are hardly any human settlements in the Sierra.

In the evaluation of waterholes, we found that NDVI had the highest *k* (0.67) for both seasons; apparently seasonality does not have an effect. This indicates that the detection of waterholes is acceptable [42, 43] in previous research. On the other hand, the *k* obtained for the NDWI improved in the wet season (*k =* 0.27) but with slight agreement [44], because it was not able to detect the other six waterholes.

Of 14 waterholes detected, desert bighorn sheep were photographed at 11 of them. There are two possible reasons why desert bighorn sheep were not recorded at the other three waterholes. First, the escape terrain was null since these waterholes were in flat sites and the lack of escape terrain may have increased the risk of predation for female herds with juveniles. For example, Berger et al. [45] reported a higher frequency of lamb kills in flat or open terrain (71%) than in steep or rugged terrain (22%); this flat or open habitat component was possibly the major reason for the sheep not using the waterholes [3, 22]. Second, the presence of domestic ungulates; for example, cattle and feral horses, can displace bighorn at some water sources [46]. Cattle or feral horses and donkeys were detected at Agua Dulce, Peralta, and San Agustin.

## Conclusions

We determined that the NDVI is more accurate than NDWI for detecting waterholes during the dry and wet seasons. Apparently the season (dry or wet) did not affect the detection of waterholes when we used the NDVI. The threshold of NDVI > 0.19 was suitable, because with this we detected 12 of 14 waterholes. We recommend a lower threshold in the dry season for the Canelo and Hemes waterholes.

The minimum detectable waterhole size using NDVI was 90.5 m^2^. We detected five *tinajas* (bedrock depressions that fill with water during the monsoon rains) during the field surveys but these are not a reliable source of water, and were not detected by the NDVI method.

## Supporting information

S1 Spreadsheet Excel spreadsheet with database of NDVI from Sentinel-2. (XLSX)

S2 Spreadsheet Excel spreadsheet with database of NDWI from Sentinel-2. (XLSX)

S3 Spreadsheet Excel spreadsheet with database of terrain escape from VRM. (XLSX)

S1 Appendix Flowchart representing the processing of satellite images and calculations of the NDVI and NDWI. (TIF)

S2 Appendix Plants identified at the periphery of waterholes detected in the Sierra Santa Isabel, Baja California. (PDF)

S3 Appendix Records of wildlife in the waterholes. (PDF)
