# Supplementary figures and images for: Waterhole detection using a vegetation index in desert bighorn sheep (Ovis canadensis cremnobates) habitat
Source: PLoS One. 2019 Jan 22;14(1):e0211202. doi: 10.1371/journal.pone.0211202 (PMC6342311; doi:10.1371/journal.pone.0211202)

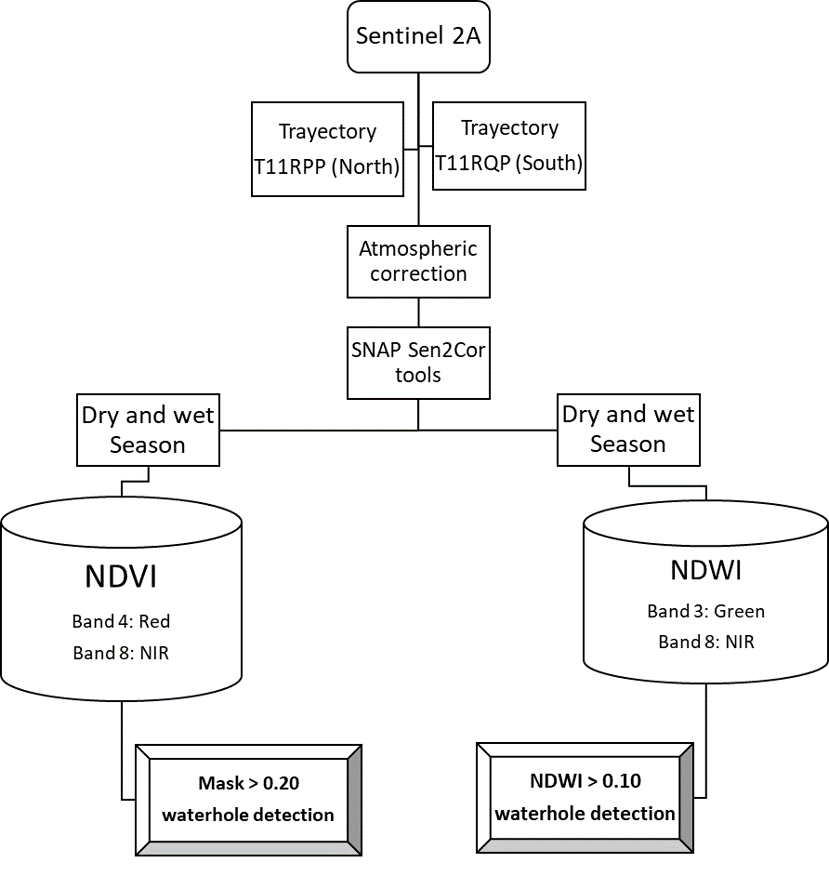

Supplement: S1 Appendix — (TIF) [file pone.0211202.s004.tif]
