# Supplementary material for: Waterhole detection using a vegetation index in desert bighorn sheep (Ovis canadensis cremnobates) habitat
Source: PLoS One. 2019 Jan 22;14(1):e0211202. doi: 10.1371/journal.pone.0211202 (PMC6342311; doi:10.1371/journal.pone.0211202)
Supplement: S2 Appendix — (PDF) [file pone.0211202.s005.pdf]

Plants identified at the periphery of waterholes detected in the Sierra Santa Isabel, Baja California.

Waterholes	Species
Matomi	*Juncus acutus, Brahea armata, Urtica dioica*
A.Grande	*Juncus acutus, Typha domingensis,*
Zamora	*Juncus acutus , Typha domingensis,*
Volcán	*Juncus acutus, Typha domingensis*
Azul	*Juncus acutus , Tamarix ramosissima*
Miramar	*Psorathamus spinosus Typha domingensis*
Palmito	*Brahea armata, Juncus acutus*
Las Blancas	*Juncus acutus, Parkinsonia microphylla*
Canelo	*Psorathamus spinosus, Olneya tesota,*
Hemes	*Juncus acutus*
Cordero	*Lupinus excubitus, Tamarix ramosissima,*
