# Supplementary material for: Waterhole detection using a vegetation index in desert bighorn sheep (Ovis canadensis cremnobates) habitat
Source: PLoS One. 2019 Jan 22;14(1):e0211202. doi: 10.1371/journal.pone.0211202 (PMC6342311; doi:10.1371/journal.pone.0211202)
Supplement: S3 Appendix — (PDF) [file pone.0211202.s006.pdf]

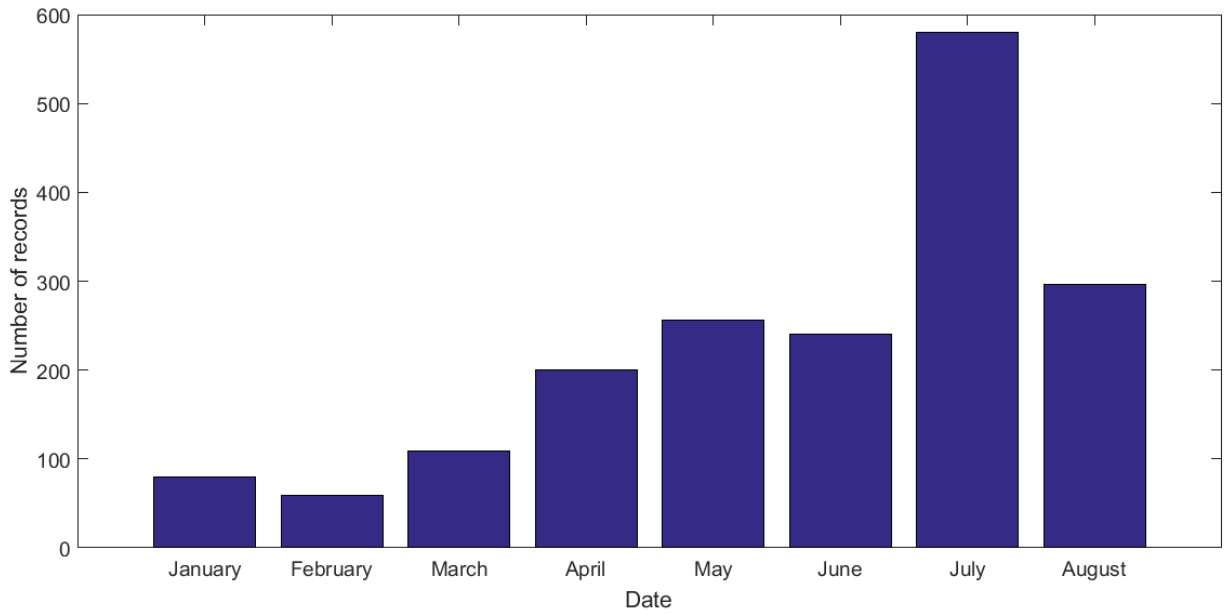

Photographic records of wildlife in all waterholes in Sierra Santa Isabel, Baja California.

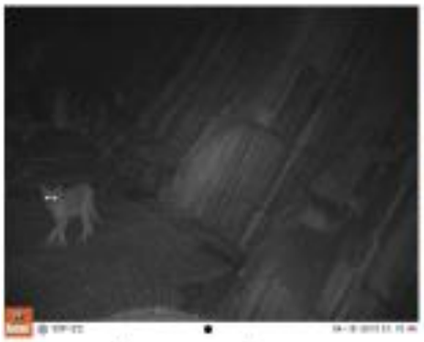

Puma concolor

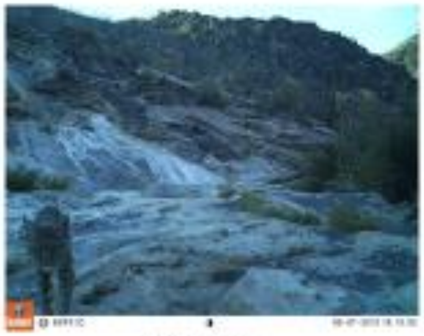

Lynx rufus

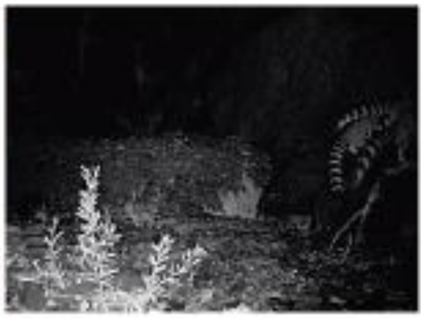

Bassariscus astuts

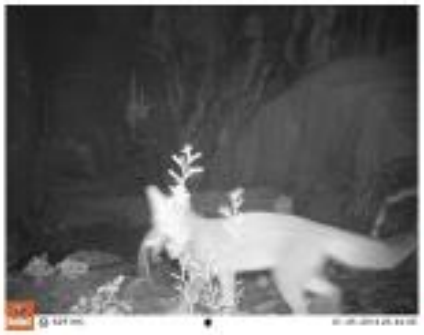

Urocyon cinereoargenteus

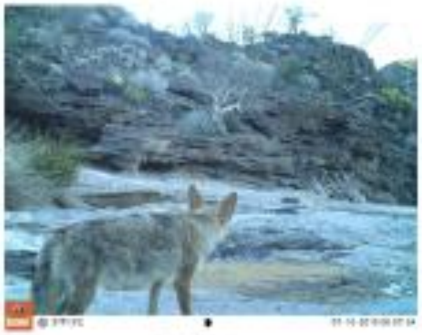

Canis latrans

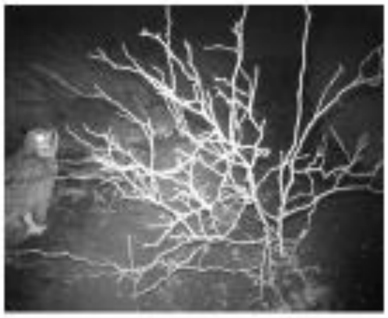

Bubo virginianus

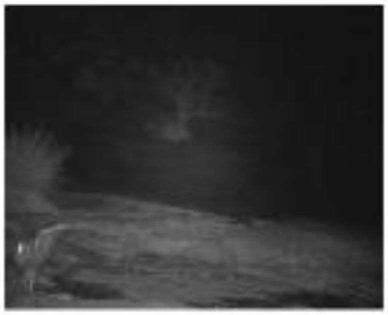

Tyto alba

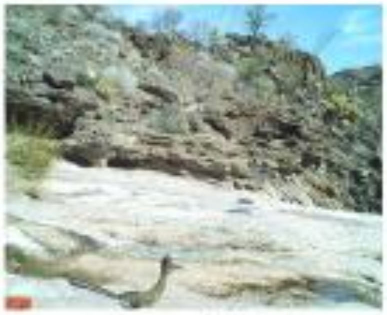

Geococcyx californicus

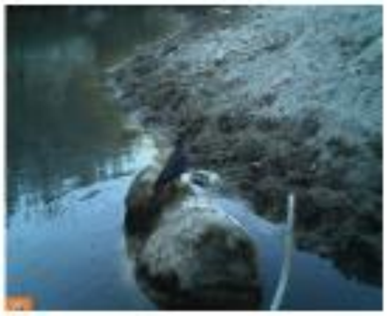

Patogioenas fasciata

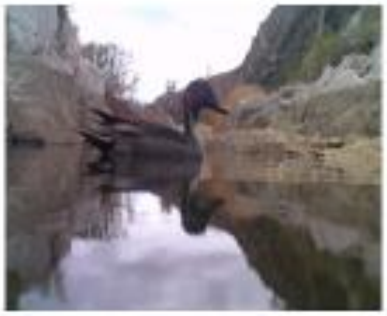

Anas acuta

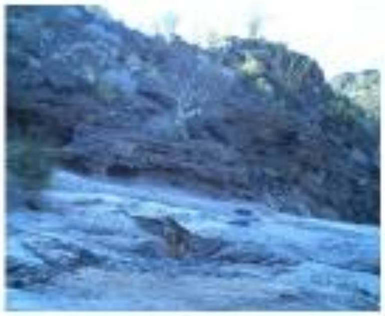

Buteo jamaicensis
